# Causal effect of molar incisor hypomineralisation on oral health-related quality of life of Australian children aged 7–16 years

**DOI:** 10.1007/s40368-025-01028-3

**Published:** 2025-04-10

**Authors:** S. Shields, T. Chen, F. Crombie, D. J. Manton, M. J. Silva

**Affiliations:** 1 Melbourne Dental School, Faculty of Medicine, Dentistry and Health Sciences, The University of Melbourne, Melbourne, Australia; 2 Inflammatory Origins, Murdoch Children’s Research Institute, Parkville, Australia; 3 Clinical Epidemiology and Biostatistics Unit, Murdoch Children’s Research Institute, Parkville, Australia; 4 Centrum voor Tandheelkunde en Mondzorgkunde, UMCG, University of Groningen, Groningen, The Netherlands; 5 Royal Children’s Hospital Melbourne, Melbourne, Australia

**Keywords:** Molar incisor hypomineralisation, Health-related quality of life, Child

## Abstract

**Purpose:**

Molar incisor hypomineralisation (MIH) is a qualitative defect of enamel characterised by demarcated opacities. Aesthetic and functional sequelae of MIH may manifest as reduced oral health related quality of life (OHRQoL). This study aims to investigate the impact of the presence and severity of MIH on children’s OHRQoL.

**Methods:**

This cross-sectional study recruited children aged 7–16 years-of-age attending specialist paediatric dental clinics in Melbourne, Australia. Clinical examination utilised the modified European Academy of Paediatric Dentistry index to quantify the presence and severity of MIH. OHRQoL data was collected via the Child Perception Questionnaire, Parent-Caregiver Perception Questionnaire and Family Impact Statement. Causal analysis used quantile regression and included poor medical health as a confounding variable. Sensitivity analysis used the same model and different strata of MIH lesion location and severity.

**Results:**

131 participants with complete self-reported OHRQoL data were included in the causal analysis. The estimated average causal effect after adjusting for poor medical health showed the estimated difference in medians of child-reported OHRQoL was 6 (CI = 2.62, 12.25, *p* = 0.02) in the MIH group compared to the unaffected group. The estimated difference in medians of self-reported OHRQoL after adjusting for poor medical health was 7 (CI = 1.87, 11.99, *p* = 0.01) for severe MIH group and − 1 (CI = − 5.16, 3.62, *p* = 0.63) for the mild group compared to those unaffected. The estimated difference in medians of self-reported OHRQoL after adjusting for poor medical health was 5.16 (CI = − 2.42, 10.99, *p* = 0.15) for participants with MIH-affected incisors compared to the rest of the cohort.

**Conclusions:**

MIH impacts children’s OHRQoL as reported by self and parent/caregiver.

**Supplementary Information:**

The online version contains supplementary material available at 10.1007/s40368-025-01028-3.

## Introduction

Molar incisor hypomineralisation (MIH) is a developmental defect of enamel defined as demarcated opacities affecting one or more first permanent molars and often permanent incisors (Weerheijm et al. 2001). The global prevalence of MIH is 13.1% (Lopes et al. 2021) and in Australia the prevalence is 14.7% (Gambetta-Tessini et al. 2018). The negative ramifications for MIH-affected children include increased dental hypersensitivity, post-eruptive breakdown (PEB), rapid development of caries lesions and poor aesthetics (Shields et al. 2024). Other adverse outcomes include reduced effectiveness of local anaesthesia for affected teeth (Rodd et al. 2007), increased treatment needs (Jälevik and Klingberg 2012) and behaviour management problems (Jälevik and Klingberg 2012, 2002). The diverse clinical presentation of MIH, varying from small areas of discolouration to profoundly sensitive PEB or tooth extraction, results in heterogeneous experiences for affected children. Management of MIH remains a challenge for clinicians who must be cognisant of the individual patient needs, and particularly the severity of the MIH, to provide effective care.

Oral health-related quality of life (OHRQoL) is a multidimensional assessment of the functional and psychosocial sequelae of oral conditions that integrates the effect on an individual’s general health and well-being (Sischo and Broder 2011). Children as young as five years of age can reliably self-report OHRQoL (Tsakos et al. 2012); however, there is a low concordance between self-assessment and OHRQoL as reported by parents/guardians (Theunissen et al. 1998). This incongruence may result from caregiver burden bias which occurs as the adults are influenced by their own emotional or physical reaction to their child’s condition (Locker et al. 2002). Parents/guardians are the chief decision makers and their OHRQoL perceptions influence treatment choices, so their input contributes to a multilayered understanding of the impact of conditions (Jokovic et al. 2003). Impacts on OHRQoL reported by children and their parents/caregivers can provide insights that facilitate more patient-centred delivery of care and subsequently improve outcomes for children.

Existing studies investigating the impact of MIH on OHRQoL have produced contradictory results, as some authors report MIH has a minimal impact on OHRQoL (Arrow 2013, 2017; de Barros et al. 2023; Dias et al. 2021; Fernandes et al. 2024; Folayan et al. 2018; Freitas Fernandes et al. 2021; Portella et al. 2018; Vanhée et al. 2022) and others report a larger impact (Dantas-Neta et al. 2016; Elhennawy et al. 2022; García-Pérez et al. 2022; Gutiérrez et al. 2019; Joshi et al. 2021; Kisacik et al. 2024; Michaelis et al. 2021; Portella et al. 2019; Reissenberger et al. 2022; Velandia et al. 2018). Methodological heterogeneity in these existing cross-sectional studies makes it difficult to compare their findings. A 2023 systematic review and meta-analysis reported children with moderate or severe MIH are 3.43 times more likely to have an impact on OHRQoL compared to those without MIH (odds ratio = 3.43, 95% CI 1.69–6.98) (Awwad et al. 2023); however, included studies had considerable heterogeneity and these results should be interpreted with caution. Many of these existing publications attempt to infer causal relationships ambiguously using observational data, but do not address the limitations of their study design and use inapt analytical methods (Schuch et al. 2023). Causal inference aims to identify and quantify the causal effect and contribute to a triangulation of evidence from multiple sources (Hernán and Robins 2020). Target trial emulation in observational dental research may lead to more valid causal conclusions by mimicking hypothetical clinical trials, which helps identify and address potential biases (Hernán et al. 2022). When causal relationships can only be established by observational research, obtaining evidence is complicated by multiple factors, including bias due to confounding (VanderWeele 2019). For cross-sectional studies, temporality can be an issue, since it is difficult to determine whether the exposure occurred before the outcome. While temporality is important for avoiding reverse causality, cross-sectional studies may provide insights into causal relationships if potential limitations and biases are adequately considered and addressed (Savitz and Wellenius 2022). There is no risk of reverse causality when seeking to evaluate the effect of MIH on oral health-related quality of life as it is implausible that OHRQoL causes MIH. However, it is necessary to assume that potential confounding variables reflect pre-existing characteristics. A cross-sectional study measuring the effect of MIH on OHRQoL at a single time point will not capture the time-varying exposure if MIH severity worsens with masticatory forces or improves with preventative strategies or dental treatment. Observational studies that examine causal relationships therefore need to ensure that the methods adopted consider bias and temporality and importantly, validation of results by replication in different studies (Hernán and Robins 2020). There is a paucity of robust causal inference examining the effect of MIH on OHRQoL and no longitudinal studies to date. Quantifying the relationship between MIH and OHRQoL is fundamental to inform clinical practice and public health interventions as it contextualises MIH within children’s general health and well-being. The objective of the present study was therefore to investigate the impact of the presence and severity of MIH on OHRQoL in a selective sample of Australian children aged 7–16 years.

## Methods

### Study design and participants

For this cross-sectional study, children aged 7–16 years attending two Australian teaching specialist paediatric dental clinics, the Royal Dental Hospital Melbourne (RDHM) and the Melbourne Dental Clinic (MDC), were recruited from September 2022 to December 2023. The RDHM is the primary public specialist dental clinic for children in the state of Victoria and eligibility to access care is means tested. MDC is a private teaching clinic associated with the University of Melbourne and there are no specific eligibility criteria other than requiring specialist-level dental care.

A total of 478 patients with appointments scheduled at the clinics were contacted via email/mail to offer the opportunity to participate in the study and informed consent was obtained for 189 individuals.

Children were excluded if they were medically compromised (ASA ≥ 2 (Abouleish et al. 2015)), syndromic, had a cleft lip and/or palate, undergoing active orthodontic treatment, non-English speaking or unable to cooperate for examination.

The present study was performed in line with the principles of the Declaration of Helsinki. Approval was granted by the ethics committee of the Royal Children’s Hospital Melbourne Human Research Ethics Committee (HREC/77810/RCHM- 2022). It is part of a large multi-centre international study (Rodd et al. 2023) investigating a different research question. The present study uses a convenience sample with a time-period limitation.

### Demographic and medical data

The parent/guardian who attended the appointment completed a questionnaire regarding the participant’s demographic and medical history. Residential postcode data were used to ascertain area-level socioeconomic disadvantage as per the Australian Bureau of Statistics’ 2021 Socio-Economic Indexes for Areas (Statistics 2021). Concession cardholder status indicates eligibility for means-tested government benefits and is another measure of deprivation in Australia (Singh et al. 2025), so these data were collected. Further details regarding these demographic variables are provided in the supplementary materials.

### Oral health-related quality of life

The Child Oral Health-Related Quality of Life (COHQoL) questionnaires (Jokovic et al. 2003; Locker et al. 2002) were used to measure OHRQoL as a continuous outcome variable and are one of the most frequently employed instruments to measure patient impact globally (Foster Page et al. 2013). They consist of a set of scales, the Child Perceptions Questionnaire, the Parental–Caregiver Perceptions Questionnaire (P-CPQ) and the Family Impact Scale (FIS) designed to capture the subjective, multifaceted experiences of children and their families.

Child-reported OHRQoL was collected via the Child Perception Questionnaire for 11–14 year olds (CPQ_11–14_) (Jokovic et al. 2002) completed by participants at the start of the study visit. The short form 16 question version of the questionnaire has been validated for use in the 7- to 16-year-old group of the study population (Foster Page et al. 2013). The eight item short forms of the P-CPQ and FIS were selected for improved feasibility and have been validated (Thomson et al. 2013). Participants and their parents/guardians were asked about the frequency of events in the previous three months in relation to children’s oral condition and answer via a Likert scale. Clinical examiners delivered the COHQoL questionnaires to participants and their parents/caregivers. The clinical examiners were registered dentists undertaking postgraduate specialisation education in paediatric dentistry at the University of Melbourne, Australia.

The Likert scales for each item were assigned a numerical value for analysis based on level of impact: ‘Never’ = 0, ‘Once or twice’ = 1, ‘Sometimes’ = 2, ‘Often’ = 3 and ‘Every day or almost every day’ = 4. P-CPQ and FIS have the response option of ‘Don’t know’ which was assigned a score of 0 (Jokovic et al. 2003). CPQ_11–14_ scores range from 0 (no impact on OHRQoL) to 64 (maximum impact on OHRQoL) and were reported via total score and at domain-level. The CPQ_11–14_ contains four domains: oral symptoms, functional limitations, emotional well-being and social well-being. P-CPQ and FIS scores range from 0 to 32; higher scores indicate poorer OHRQoL for each of the scales. There are two global questions for both the CPQ_11–14_ and the P-CPQ and FIS which can be used for internal validation of the instruments to evaluate outcome misclassification bias.

OHRQoL data were excluded from analysis if there were item non-responses or duplicate answers.

### Clinical data

Clinical examination utilised the modified European Academy of Paediatric Dentistry MIH (Ghanim et al. 2019, 2017; Lygidakis et al. 2022) and International Caries Detection and Assessment System (ICDAS) (Ismail et al. 2007) indices to quantify the presence and severity of MIH and caries lesions. Participants were diagnosed with MIH when at least one first permanent molar was affected with a demarcated opacity greater than 1 mm in size, post-eruptive breakdown, atypical caries lesion, atypical restoration or was missing due to MIH. The ICDAS index has a two-stage diagnostic process for each tooth surface: first to classify if it is sound, sealed, restored or missing and second to classify the carious lesion status. The carious lesion status is diagnosed as either sound code 0, visual change in enamel when air dried code 1, visual change in enamel when examined wet code 2, localised enamel carious breakdown code 3, dark underlying shadow from dentine with/out enamel breakdown code 4, cavitation with visible dentine code 5 or extensive dentine cavitation involving at least half of the surface code 6. Data collection involved a total of ten trained and calibrated clinical examiners with kappa scores for MIH intra-examiner and inter-examiner reliability ranging from 0.77 to 0.94 and 0.52 to 0.96, respectively. Kappa scores for ICDAS intra-examiner and inter-examiner reliability ranged from 0.66 to 0.95 and 0.71 to 0.89, respectively.

### Statistical analysis

Data management software *REDCap* (Harris et al. 2019, 2009) (version 13.10.6) was used for secure digital storage, which preserves data hygiene with an automatic log of any changes. Analysis was undertaken using statistical software *R* (R Core Team 2020).

Descriptive statistics were reported as means with standard deviation (SD) for symmetrical variables and medians with interquartile range (IQR) for skewed variables. Sample characteristics were stratified by exposure as presence of MIH. Descriptive subgroup statistics were reported for OHRQoL across different strata of MIH lesion location and severity. MIH was classified as severe if one or more index teeth were affected with post-eruptive breakdown, atypical caries lesion, atypical restoration or extracted due to MIH. Individuals were assigned to the mild MIH subgroup if there were no teeth affected by severe MIH. As there is no international consensus regarding MIH phenotypic classification another subgroup variable, based on MIH lesion location, was created for MIH-affected individuals indicating incisor involvement as aesthetically compromised anterior teeth could impact OHRQoL (Leal et al. 2017).

**Fig. 1:**
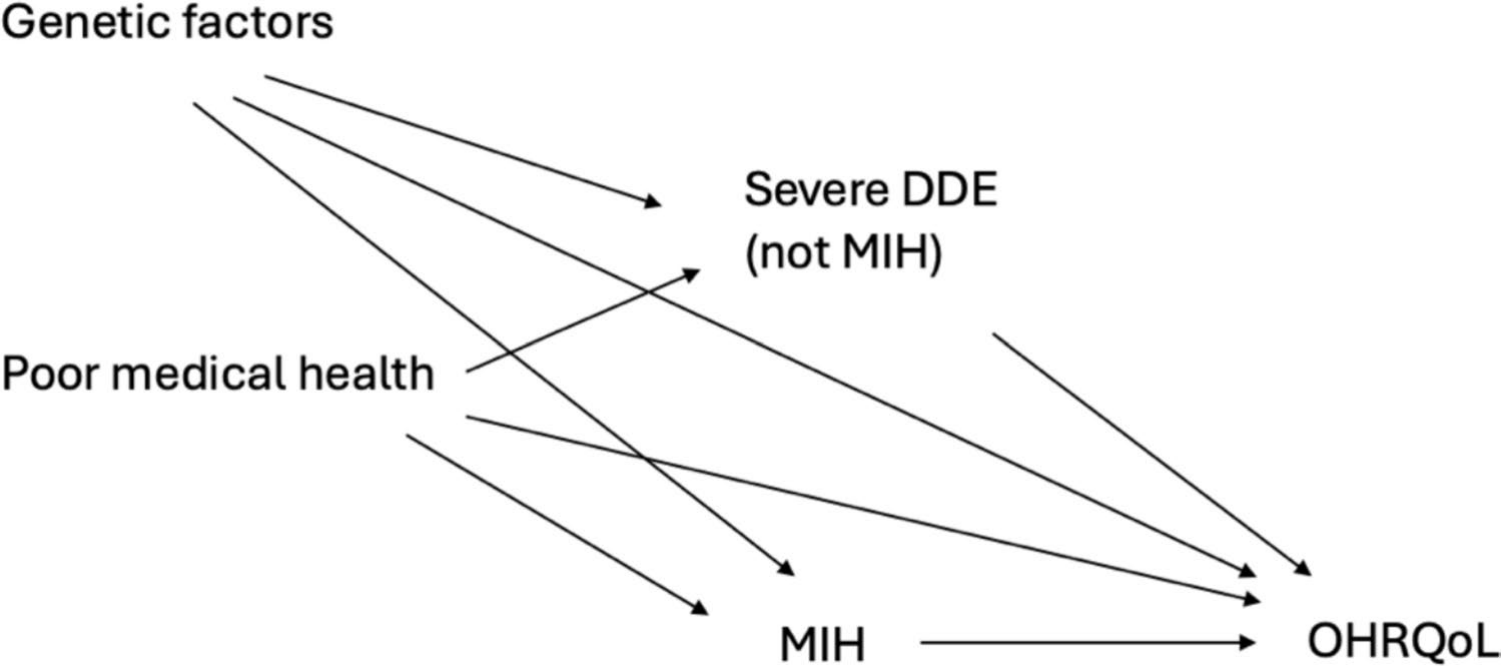
Representative direct acyclic graph (DAG) for statistical model

Medical history data were itemised as per the World Health Organization’s International Classification of Disease (ICD) *ICD- 10 Version:2019* (World Health Organization 2004) coding. Conditions associated with MIH aetiology (Garot et al. 2018; Silva et al. 2016) were incorporated as a confounding variable (VanderWeele 2019) ‘poor medical health’. Genetic factors, although currently unknown, may contribute to MIH aetiology and impact OHRQoL so are an unmeasured confounding variable. Developmental enamel defects (DDE) other than MIH were classified based on clinical presentation into mild and severe. Severe DDE, including amelogenesis imperfecta, anterior opacities, severe chronological hypoplasia and severe fluorosis, could share common causes with MIH and effect OHRQoL. Participants were excluded if severe DDE other than MIH were present as they are a source of confounding bias. Mild DDE including localised mild hypoplasia, posterior opacities and mild fluorosis were incorporated in the analysis. These mild defects are not confounding variables as their impact on OHRQoL is negligible and they are representative of a comparison group. The directed acyclic graph (DAG) outlining the assumptions about these relationships is detailed in Fig. 1.

Quantile regression was used to estimate the impact of MIH presence or absence on OHRQoL, as measured by continuous CPQ_11–14_ scores, adjusting for the confounding variable of poor medical health. Sensitivity analysis was conducted to investigate how the effect varies between different levels of MIH severity (absent, mild or severe). A separate statistical model included the incisor subgroup versus the rest of the cohort (participants with MIH but no incisor involvement and those unaffected) using quantile regression and adjusting for the confounding variable of poor medical health. All statistical models included age, which is only associated with the outcome as a covariate to improve the precision (Benkeser et al. 2020). Confidence intervals and p values were calculated via the bootstrap method (Efron 1979).

Caries lesions are on the causal pathway from MIH to OHRQoL and are therefore a mediating variable (Albert and Nelson 2011). They are not a confounding variable in the relationship between MIH and OHRQoL as caries lesions do not contribute to MIH aetiology. The aim of this paper is to estimate the overall causal effect of MIH on OHRQoL, and it is only meaningful to investigate the influence of caries lesions via causal mediation analysis once a sufficient total causal effect of MIH on OHRQoL has been observed.

## Results

A total of 151 participants attended for data collection and there were complete data available for self-reported and parent/caregiver-reported OHRQoL for 131 and 116 participants, respectively. The socio-demographic details, clinical findings and OHRQoL data are detailed in Table 1.

**Table 1:** Characteristics of study participants

	Total sample	MIH	
		Present	Absent
*n* (%)	131 (100)	44 (33.6)	87 (66.4)
Age: years Mean (SD)	10.3 (2.3)	10.3 (2.1)	10.5 (2.4)
Sex males: * n* (%)	73 (55.7)	22 (50.0)	51 (58.6)
Sex females: * n* (%)	58 (44.3)	22 (37.9)	36 (62.1)
Medical history with MIH aetiology: * n* (%)	21 (16.0)	8 (18.2)	36 (81.8)
Concession cardholder status: * n* (%)	102 (77.9)	38 (86.4)	64 (73.6)
Area-level socioeconomic disadvantage: mean (SD)	993.9 (62.0)	1003 (63.0)	990 (61.3)
CPQ_11–14_ median [IQR]	14 [9, 20.5]	18 [11, 24]	12 [8, 18]
P-CPQ median [IQR]	6 [2, 12] *n* = 116	8 [3, 13] *n* = 41	5 [2, 9.5] *n* = 75
FIS median [IQR]	2 [0, 6.3] *n* = 116	2 [0, 9] *n* = 41	1 [0, 4] *n* = 75

*n* = number of participants, SD = standard deviation, IQR = interquartile range, MIH = molar incisor hypomineralisation, CPQ_11–14_ = child perception questionnaire, P-CPQ = parental–caregiver perception questionnaire, FIS = family impact statement

Of the 131 participants included in the causal analysis 55.7% (*n* = 73) were male and the mean age was 10.3 (SD 2.3) years. History of medical conditions associated with MIH aetiology were reported by 16.0% (*n* = 21) of individuals. The distribution of the socioeconomic status of participants was skewed as 77.9% (*n* = 102) of participants were concession cardholders and area-level socioeconomic deprivation was greater than the Australian average (Statistics 2021). The prevalence of MIH was 33.6% (*n* = 44) with incisor involvement in 59.1% (*n* = 26) of affected children. Over two-thirds of MIH-affected participants had teeth with severe MIH (68.1%, *n* = 30). Over half (55.7%, *n* = 73) of participants had previous caries experience and clinical caries lesions (ICDAS > 0) present in 67.9% (*n* = 89) of all children and extensive caries lesions (ICDAS ≥ 5) present in 35.1% (*n* = 46).

### Oral health-related quality of life

**Fig. 2:**
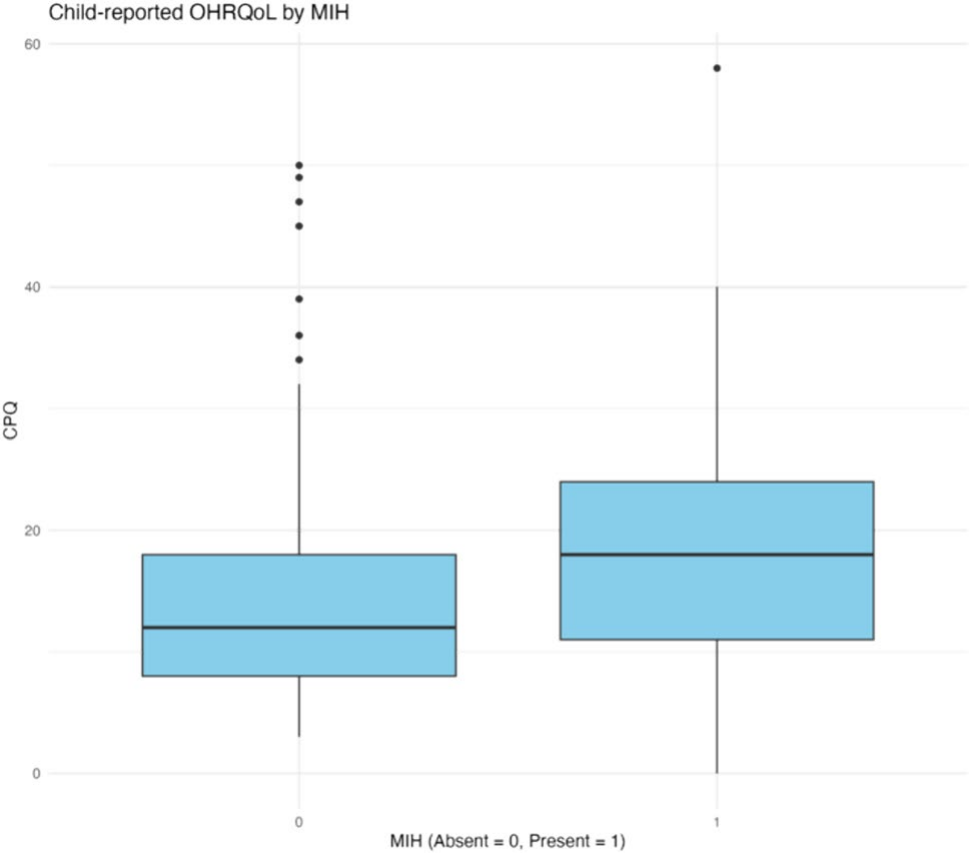
Comparison of child-reported OHRQoL by MIH

**Fig. 3:**
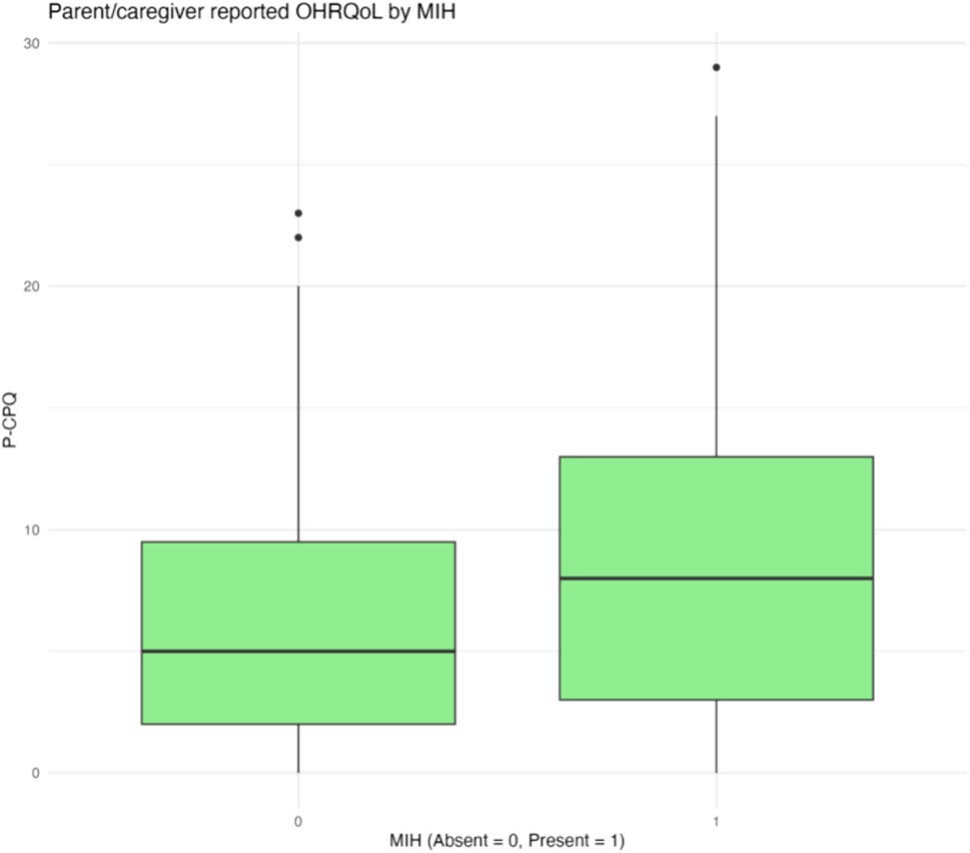
Comparison of parent/guardian-reported OHRQoL by MIH

Data relating to OHRQoL as per the CPQ_11–14_, P-CPQ and FIS had a skewed distribution, and are reported as median total scores in Table 1 and Figs. 2 and 3. Higher scores indicate a poorer OHRQoL. Overall, the study population’s child-reported OHRQoL was media* n* = 14 (IQR 9, 20.5), with median P-CPQ = 6 (IQR 2, 12) and median FIS = 2 (IQR 0, 6.3).

Participants with MIH had poorer child-reported OHRQoL (median CPQ_11–14_ = 18, IQR = 11, 24) compared to those unaffected by MIH (median CPQ_11–14_ = 12, IQR = 8, 18). Parent/guardian-reported OHRQoL was also poorer for children with MIH (median P-CPQ = 8, IQR = 3, 13) compared to those unaffected by MIH (median P-CPQ = 5, IQR = 2, 9.5). The difference on the family scale was nominal between the MIH groups.

**Fig. 4:**
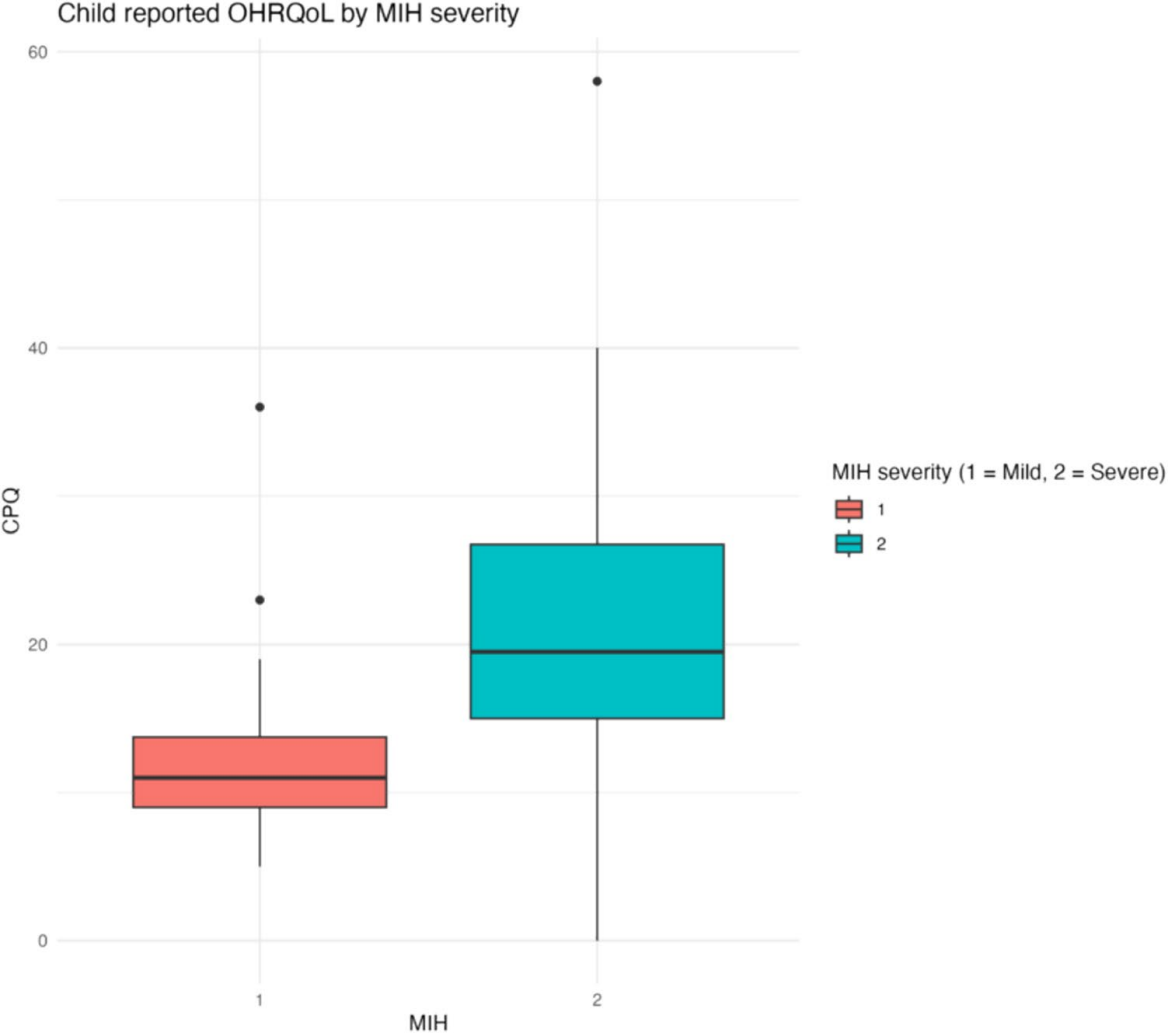
Comparison of child-reported OHRQoL by MIH severity level

**Fig. 5:**
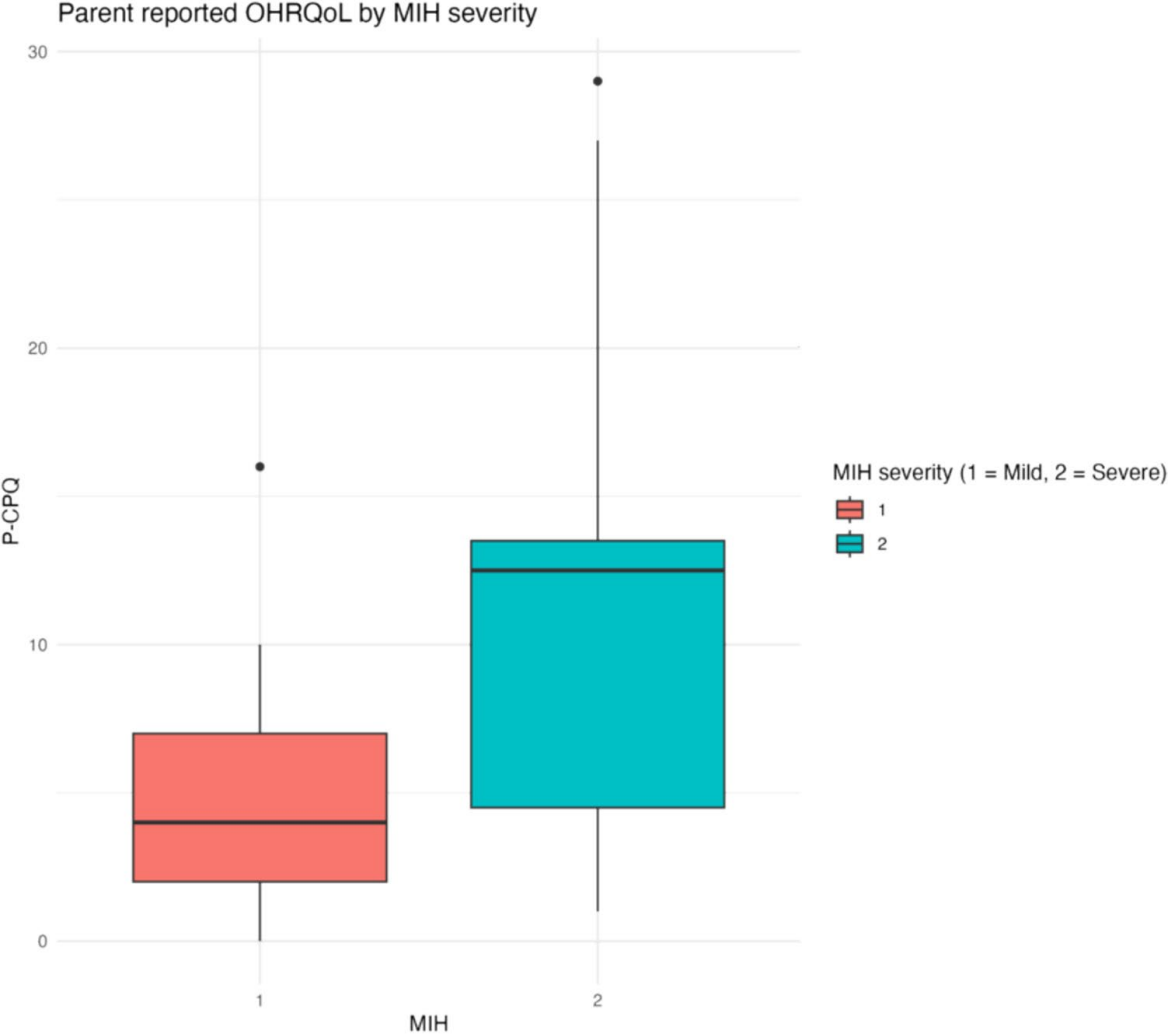
Comparison of parent/guardian-reported OHRQoL by MIH severity level

Descriptive subgroup statistics for MIH severity and location are detailed in Table 2 with outcomes reported for each COHQoL instrument. Figures 4 and 5 show the distribution of CPQ_11–14_ and P-CPQ by MIH severity level (see the supplement for further detail regarding domain-level reporting for the CPQ_11–14_).

**Table 2:** MIH subgroup results—median [IQR]

	MIH	Mild MIH, no severely affected teeth	Severe MIH	MIH with incisor involvement
CPQ_11–14_	18 [11, 24] *n* = 44	11 [9, 13.8] *n* = 14	19.5 [15, 26.8] *n* = 30	18.5 [12, 29.8] *n* = 26
P-CPQ	8 [3, 13] *n* = 41	4 [2, 7] *n* = 13	12.5 [4.5, 13.5] *n* = 28	8.5 [4.3, 15.2] *n* = 24
FIS	2 [0, 9] *n* = 41	0 [0, 2] *n* = 13	6.5 [1.8, 10.2] *n* = 28	2 [0.8, 10] *n* = 24

*n* = number of participants, SD = standard deviation, IQR = interquartile range, MIH = molar incisor hypomineralisation, CPQ_11–14_ = child perception questionnaire, P-CPQ = parental–caregiver perception questionnaire, FIS = family impact statement

### Causal effect of MIH on OHRQoL

The estimated average causal effect adjusting for poor medical health showed the estimated difference in medians of child-reported OHRQoL was 6 (CI = 2.62, 12.25, *p* = 0.02) in the MIH group compared to the unaffected group (Table 3). The estimated difference in medians of self-reported OHRQoL was 7 (CI = 1.87, 11.99, *p* = 0.01) for severe MIH and − 1 (CI = − 5.16, 3.62, *p* = 0.63) for the mild group compared to those unaffected (Table 3). The estimated difference in medians was 5.16 (CI = − 2.42, 10.99, *p* = 0.15) for participants with MIH-affected incisors compared to the rest of the cohort.

**Table 3:** Causal effect of MIH on OHRQoL

	Difference in median CPQ_11–14_ relative to participants with no MIH	Confidence interval (95%)	*P* value
MIH	6	2.62, 12.25	0.02
Mild MIH	− 1	− 5.16, 3.62	0.63
Severe MIH	7	1.87, 11.99	0.01

MIH = molar incisor hypomineralisation, CPQ_11–14_ = child perception questionnaire. Estimated difference in median CPQ_11–14_ from quantile regression with poor medical history as confounding variable and covariate of participant’s age

## Discussion

In the present study the presence of MIH leads to poorer self-reported OHRQoL compared to unaffected participants. Subgroup causal analysis of children with severe MIH or incisor involvement also demonstrated an increase in average effect on OHRQoL compared to the non-MIH group. However, the causal effect estimates should be interpreted with caution as there is low precision with a wide confidence interval that may be secondary to the study’s sample size. This result is in accordance with existing literature that demonstrates a greater impact on OHRQoL for MIH-affected children (Gutiérrez et al. 2019) and particularly for individuals with severe MIH (Dantas-Neta et al. 2016; Elhennawy et al. 2022; García-Pérez et al. 2022). The present differences in median CPQ_11–14_ are greater than other investigations of children with high caries experience (Foster Page et al. 2019) or severe malocclusion (Scapini et al. 2013) and is commensurate to increased CPQ_11–14_ scores reported by children with cleft lip and/or palate compared to unaffected individuals (Aleksieva et al. 2021). Methodological heterogeneity makes comparison with other OHRQoL studies challenging, particularly as their outcome is reported as a mean, whereas the present study reported median OHRQoL as the outcome was skewed. Participants with MIH-affected incisors demonstrated poorer OHRQoL versus a comparison group of those unaffected and individuals with MIH but no incisor involvement; however, there was a wide confidence interval that included the null. This result should be interpreted with caution, but it highlights the importance of considering the incisor involvement in the future investigations of OHRQoL and more broadly the MIH phenotypic classification. Parent/caregiver-reported OHRQoL was found to be poorer in children with MIH, but causal analysis was not undertaken. The present results demonstrate that there may be a causal relationship and particularly that severe MIH likely impacts OHRQoL, with the aforementioned caveat that clearly justifies the need for further studies.

As MIH is a developmental condition it is impossible to undertake randomisation to investigate knowledge gaps and therefore observational causal inference is crucial to provide further insights. Causal inference with observational data is advantageous as unique evidence obtained from real-world settings can direct future experimentations or evaluate the everyday generalisability or applicability of estimated causal relationships or interventions (Dahabreh and Bibbins-Domingo 2024). A strength of the present study is the clearly articulated analysis plan adhering to the framework for observational causal inference (Dahabreh and Bibbins-Domingo 2024) with identification of potential bias and limitations. The present study is the first to estimate the average causal effect of MIH on OHRQoL with this methodological rigour, and while the findings are not new, the methods are novel and provide important validation of weaker evidence that has not adopted the robust methods that are recommended in causal inference. Accurate identification and adjustment of confounding variables is essential for unbiased causal estimates in observational research (Schuch et al. 2023), and no existing study investigating the effect of MIH on OHRQoL correctly mitigated confounding bias. This analysis included medical conditions associated with MIH aetiology as a confounding variable as per the DAG (see Fig. 1). MIH aetiology is currently unknown and is likely multifactorial with contribution from genetics, epigenetics, pre-natal and peri-natal environmental exposures and early childhood illnesses (Garot et al. 2022; Silva et al. 2016). Genetic factors were detailed in the DAG as they could contribute to the aetiology of both MIH and other DDE, and therefore other severe DDE were excluded to address this putative confounding bias. The unknown and unmeasured genetic and environmental exposures that could contribute to MIH aetiology are a residual source of potential confounding bias. Missing OHRQoL data are another limitation of the study that could introduce selection bias. Data collection for the present study was conducted in accordance with its study protocol; however, there is currently no recognised formal training for OHRQoL data collection, and a more formalised training protocol could limit missing data from item non-response and duplicate responses in future studies. As there are no OHRQoL instruments validated for use in MIH populations, measurement bias may be present if the COHQoL questionnaires do not accurately capture the experiences of MIH-affected children. Another limitation of the present study is the cross-sectional design, so the temporal sequence between exposure and outcome cannot be established. Fortunately, there is no risk of reverse causality, a critical consideration for causal inference in cross-sectional studies, as OHRQoL does not cause MIH. As there are currently no longitudinal studies examining the effect of MIH on OHRQoL, valuable causal insights may be obtained from a cross-sectional study.

A major strength of the present study is the high proportion of participants with socioeconomic deprivation, as these individuals are more difficult to engage in research within high income countries such as Australia (Bonevski et al. 2014). Equitable research is paramount to improve the understanding of the socioeconomic determinants of oral health disparities and inform appropriate strategies (Do et al. 2010). The sample population was children accessing specialist paediatric dental care, and therefore, the findings are only generalisable to this population. Extending the present study’s conclusions to the general population would need stronger assumptions and is not appropriate. Further longitudinal studies investigating the OHRQoL of children affected by MIH should utilise population-based samples of a larger size to improve precision of the effect estimate.

## Conclusion

MIH impacts children’s OHRQoL as reported by self and parent/caregiver in this cross-sectional study of children aged 7–16 years attending two teaching specialist paediatric dental clinics in Melbourne, Australia. Participants with severe MIH or incisor involvement also demonstrated an increase in average effect on OHRQoL compared to the non-MIH group.

## Supplementary Information

Below is the link to the electronic supplementary material.

Supplementary file1 (DOCX 15 KB)

## Data Availability

Given the ethics requirements of this study, the data that support the findings of this study are not openly available. The R code is available from the corresponding author upon reasonable request. Data are located in controlled access data storage at Murdoch Children’s Research Institute.
