# Supplementary material for: Causal effect of molar incisor hypomineralisation on oral health-related quality of life of Australian children aged 7–16 years
Source: Eur Arch Paediatr Dent. 2025 Apr 10;26(5):903–13. doi: 10.1007/s40368-025-01028-3 (PMC12532629; doi:10.1007/s40368-025-01028-3)
Supplement: Supplementary file 1 — Supplementary file1 (DOCX 15 KB) [file 40368_2025_1028_MOESM1_ESM.docx]

**SUPPLEMENTARY MATERIAL**

**Australian demographic data**

The 2021 Census of population and housing from the Australian Bureau of Statistics informs the Australian Socio-Economic Indexes for Areas (SEIFA) by merging data, such as income, education and housing, to summarise the socioeconomic characteristics of an area (Australian Bureau of Statistics 2021). After analysis the SEIFA scores are rescaled to a mean of 1,000 and standard deviation of 100 for intuitive interpretation (Australian Bureau of Statistics 2021). SEIFA has 4 indexes including the Index for Relative Socioeconomic Disadvantage (IRSD) which was used in this study.

IRSD are composite scores for small area-level socioeconomic deprivation, they don’t directly reflect the relative disadvantage of an individual residing in that postcode. As scores are created on an arbitrary scale, they also do not represent the quantity of disadvantage i.e. an area with a score of 500 is not twice as disadvantaged as an area with a score of 1000. Residential postcodes from participants were matched to the IRSD scores and summarised for each exposure of interest to this study. IRSD scores below 1,000 should be interpreted as greater socioeconomic deprivation than the average Australian.

Concession cards are allocated by the Australian federal government to an individual who receives a specific government payment, pension or allowance (Singh et al. 2025). These include age pension, carers pension, disability allowance, disability support pension, family allowance, family payment, JobSeeker payment, low-income allowance, mobility allowance, Newstart, partner allowance, parenting payment, parenting/partner allowance, sickness allowance, special benefit, widow allowance, wife pension and widow pension. Concession card eligibility is therefore a marker of socioeconomic disadvantage. Individuals who hold concession cards in Australia have increased access to public health services including dentistry.

**Domain level reporting of OHRQoL as per each MIH subgroup**

Table 5: CPQ_11-14_ domain level reporting via MIH subgroup – median [IQR].

|  | Oral symptoms | Functional limitations | Emotional well-being | Social well-being |
| --- | --- | --- | --- | --- |
| Overall  n = 131 | 5 [4, 7] | 3 [1, 5] | 1 [0, 4] | 1 [0, 3] |
| MIH  n = 44 | 6 [4, 8.3] | 3 [1, 5.3] | 2 [0, 5] | 1 [0, 3] |
| Mild MIH, no severely affected  n = 14 | 5.5 [4, 6] | 2 [1, 3.8] | 0.5 [0, 1] | 1 [0, 1.8] |
| Severe MIH  n = 30 | 7 [4.3, 9] | 3 [2, 6] | 4 [0, 6] | 2 [0,3] |
| MIH incisors  n = 26 | 6 [4.3, 9] | 3 [2, 7.5] | 4 [0.3, 6] | 1.5 [0, 3] |

n = number of participants, SD = standard deviation, IQR = interquartile range, MIH = molar incisor hypomineralisation, CPQ11-14 = child perception questionnaire
